# Ganglion Cysts in the Hallux: A Report of Two Cases

**DOI:** 10.7759/cureus.54423

**Published:** 2024-02-18

**Authors:** Hirotaka Fukushima, Tadashi Kimura, Mitsuru Saito, Makoto Kubota

**Affiliations:** 1 Department of Orthopaedic Surgery, The Jikei University School of Medicine, Tokyo, JPN

**Keywords:** hallux ganglion, flexor hallucis longus, tendon sheath, communication stalks, recurrence

## Abstract

We experienced two cases of intractable hallux ganglion. Case 1 was a 70-year-old woman with a recurrent ganglion and severe pain on the plantar aspect of the hallux. The continuity between the mass and the distal flexor hallucis longus (FHL) tendon sheath was confirmed. The ganglion was resected along with part of the tendon sheath, and the tendon sheath was incised as proximally as possible. Case 2 was a 69-year-old woman with a ganglion on the dorsal aspect of the interphalangeal joint that repeatedly ruptured due to thinning of the skin. The ganglion was contiguous with the joint capsule but not with the FHL tendon sheath, and the entire capsule was resected. There was no recurrence one year after surgery in either case. The risk of recurrence of an intractable hallux ganglion can be reduced by blocking the synovial supply route and lowering the pressure inside the joint or tendon sheath.

## Introduction

Ganglions are benign cystic lesions containing gelatinous material, commonly arising from the joint capsule, tendon sheath, synovium, or bursa, the majority of which are asymptomatic [1]. However, ganglions of the lower extremities are larger in size than those occurring in the upper extremities and are often symptomatic. Hallux ganglions are particularly susceptible to shoe irritation, which can cause problems such as tightness and severe pain due to the mass, neuropathy due to pressure, and self-destruction, which can often be clinically problematic [2-3]. The basic treatment is conservative, including observation, compression, aspiration of cyst fluid, corticosteroid injection, and sclerotherapy, but the recurrence rate is as high as 33% to 63% [3]. Surgical resection must be chosen when they resist conservative treatment, but the recurrence rate after resection of all foot ganglia is still high, ranging from 5% to 30% [4]. In this report, we present two cases of persistent hallux ganglion cysts.

## Case presentation

Case 1

**Figure 1:**
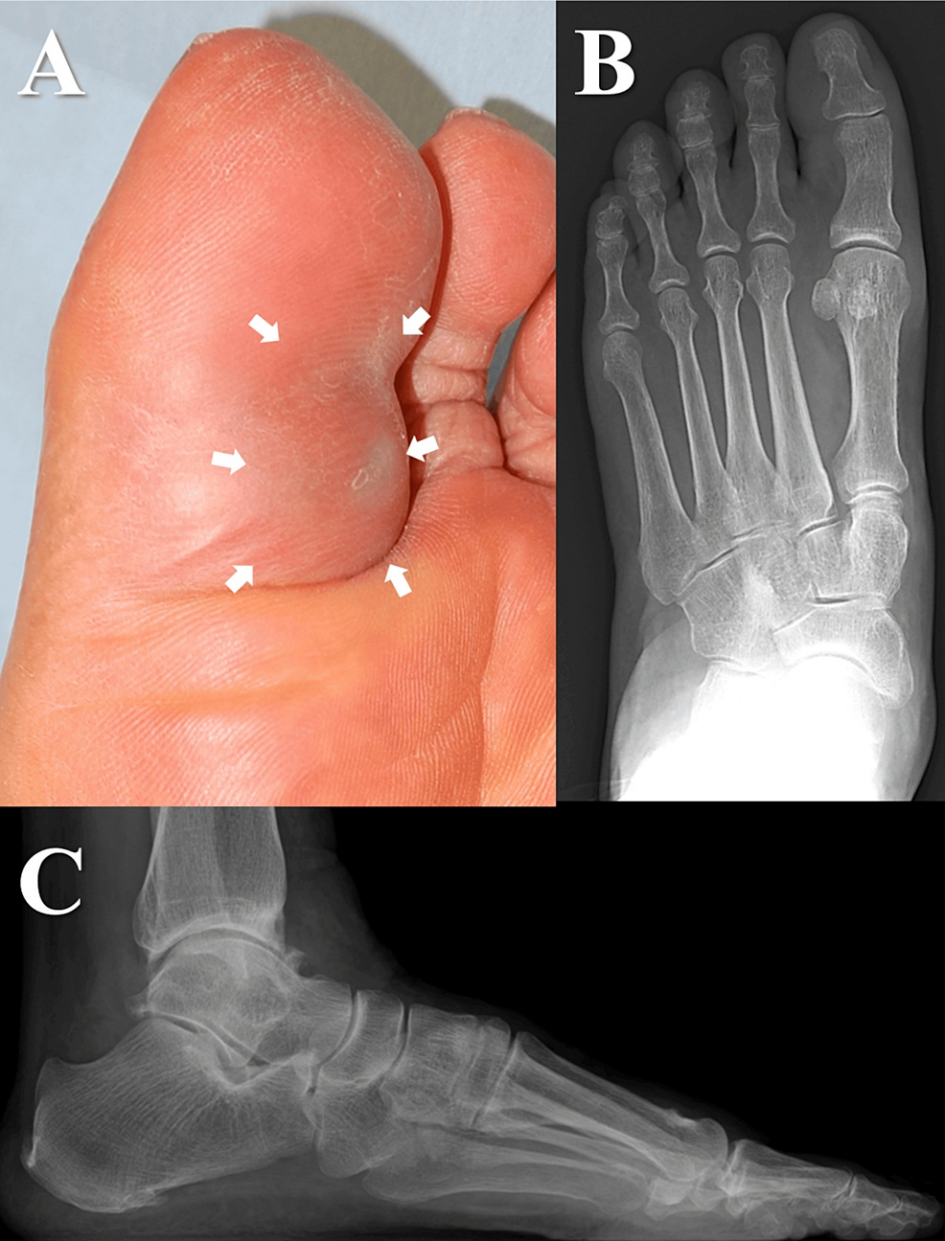
Preoperative clinical photograph and radiographic findings (Case 1) (A) A tender, elastic, firm, and mobile swelling is identified on the plantar aspect of the hallux. (B and C) Anteroposterior and lateral radiographs of the left foot show no remarkable changes.

A 70-year-old woman complained of pain in walking due to a painful enlarging mass, present for three months, on the plantar surface of her left hallux. Despite undergoing three rounds of aspiration for a ganglion diagnosed at another hospital, she was referred to our department for worsening pain and numbness. Physical examination revealed a tender, elastic, firm, and mobile subcutaneous tumour measuring 3 × 2 cm on the plantar aspect of the proximal phalanx and extending to the distal end of the lateral aspect of the hallux. The sensation was impaired on the lateral plantar aspect of the interphalangeal (IP) joint, and Tinel’s sign was positive (Figure 1A). Plain radiographs (Figure 1B-1D) showed ankle bone spur formation and joint space narrowing due to osteoarthritis.

**Figure 2:**
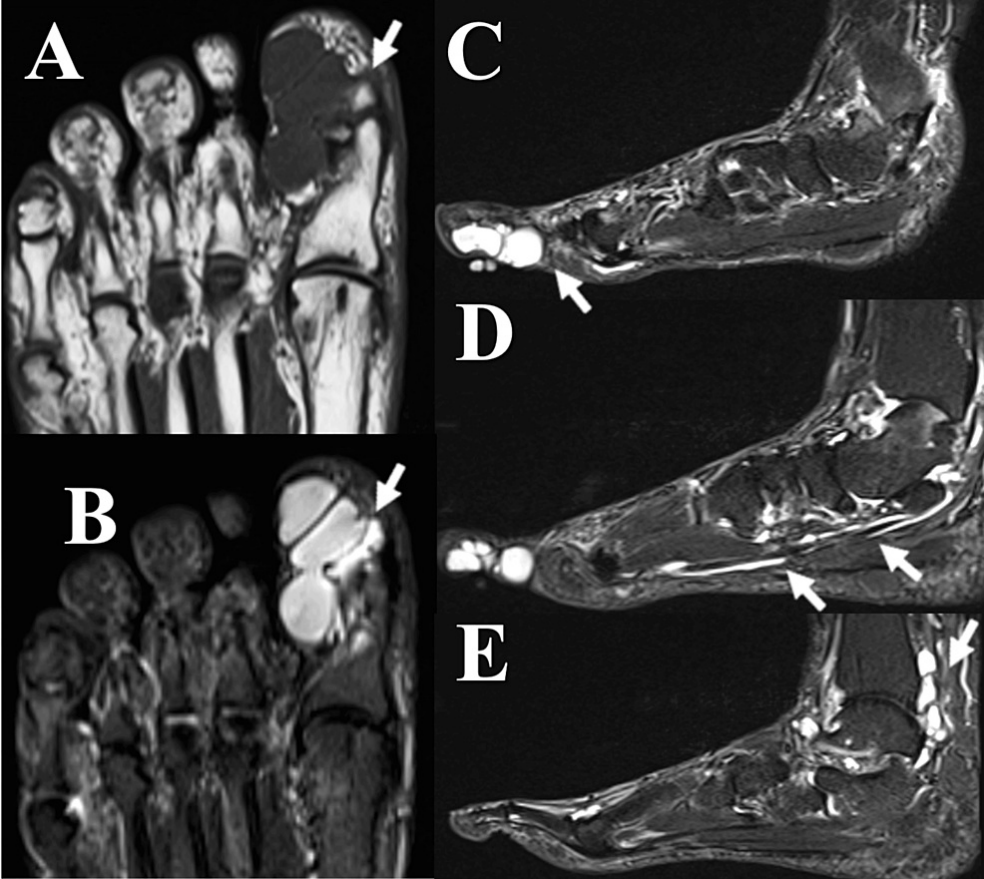
Findings on MRI (Case 1) (A-B) Axial T1-weighted (upper) and short tau inversion recovery (lower) sequences show a multi-cystic nodular lesion on the plantar aspect of the hallux. (C–E) MRI of the left ankle demonstrating short tau inversion recovery sequences in the sagittal plane. Along with the cysts, fluid accumulation was observed within the FHL tendon sheath and ankle joint. MRI: magnetic resonance imaging, FHL: flexor hallucis longus

The MRI revealed hypointense cysts on T1-weighted images and hyperintense cysts on T2-weighted images. The diagnosis was ganglion cysts with fluid accumulation within the flexor hallucis longus (FHL) tendon sheath extending from the hallux to the foot and ankle joint (Figure 2).

**Figure 3:**
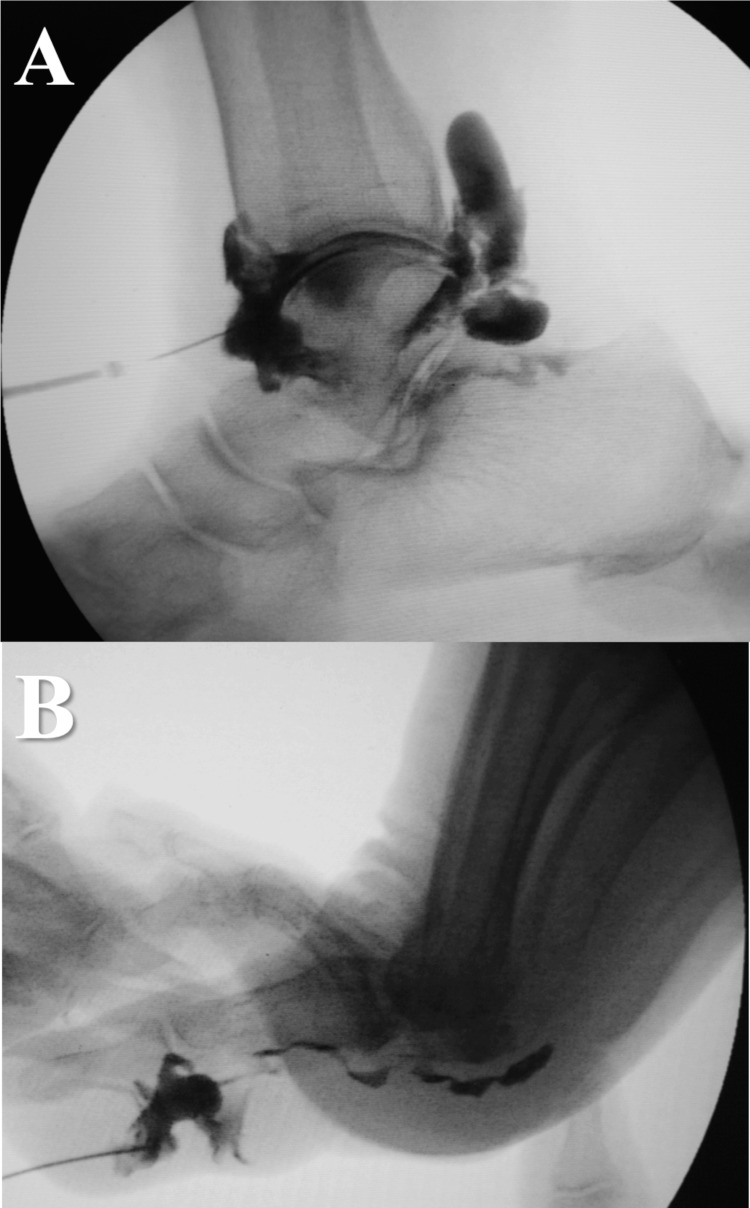
Findings on arthrography (Case 1) (A) Injection into the ankle joint revealed the FHL tendon sheath, which extended to the posterior aspect with cystic lesions. (B) Injection into the cysts revealed the FHL tendon sheath, which extended to the plantar aspect. There was no continuity between the cysts and the ankle joint. FHL: flexor hallucis longus

Due to recurrent and painful hallux ganglion cysts despite multiple aspirations, communication with the FHL tendon sheath and synovial fluid supply from the ankle joint was suspected, and surgical excision was performed. Arthrography detected communicating tracts between the ankle joint, FHL tendon sheath, and cystic lesion. A radiopaque contrast agent injected into the ankle joint leaked into the FHL tendon sheath with the cyst in the posterior ankle joint, but no further communication was found. Contrast injected into the ganglion reached the base of the metatarsal head but did not enhance the proximal lesion. The FHL tendon sheath did not communicate with the metatarsophalangeal (MTP) or IP joints (Figure 3).

**Figure 4:**
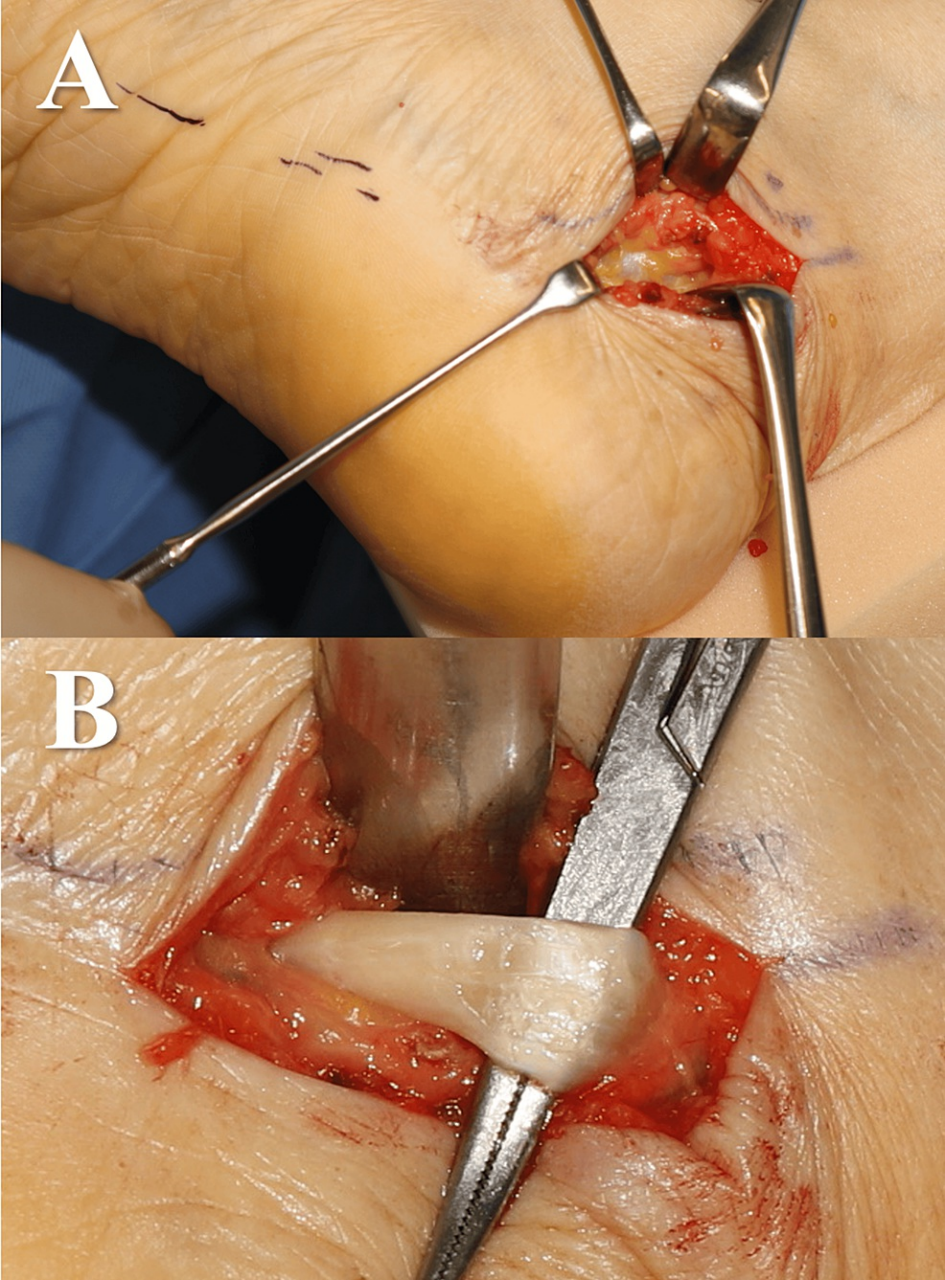
Intraoperative photographs (Case 1) (A) When the area around the subtalar joint was dissected, significant fluid correction was observed in the FHL tendon sheath. (B) When the FHL tendon sheath was incised, a large amount of synovial fluid was released. There were no abnormal findings in the tendon. FHL: flexor hallucis longus

**Figure 5:**
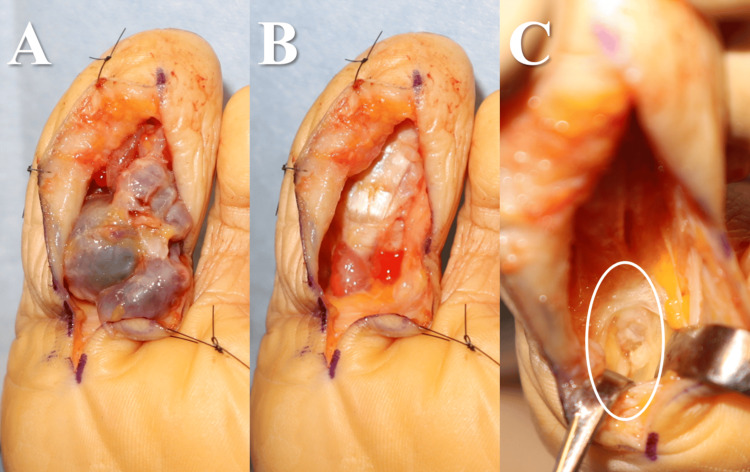
Intraoperative photographs (Case 1) (A-B) Partial continuity was observed between the tumour and the FHL tendon sheath, and part of the sheath was resected along with the tumour. (C) The sheath was opened as proximally as possible. FHL: flexor hallucis longus

An incision was made near the posterior medial malleolus, and the FHL tendon sheath at the posterior part of the talocalcaneal joint was removed with the drainage of a large amount of synovial fluid. There were no specific findings in the tendon (Figure 4), and manual compression of the ganglion cysts did not result in further drainage of synovial fluid. A zig-zag incision was made beyond the ganglion cysts, and the cysts were then excised from the hallux. The en bloc resection was performed due to the difficulty of detaching the cysts from the tendon sheath at the base of the tendon. After the excision of the FHL tendon sheath as proximal as possible, a large amount of synovial fluid was drained, indicating successful release (Figure 5).

**Figure 6:**
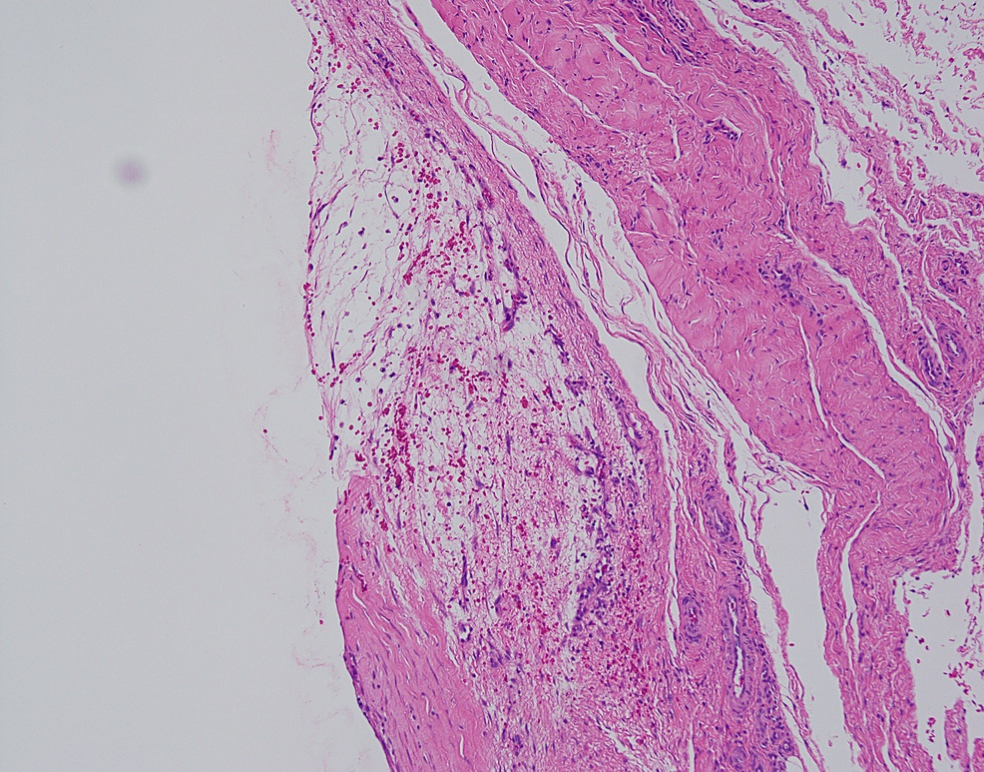
Histopathological analysis (Case 1) A ganglion cyst consists of a thin collagenous fibrous wall filled with degenerated mucus, capillary hyperplasia, and hemosiderin deposition.

Histopathological analysis confirmed a ganglion consisting of a thin collagenous fibrous wall filled with degenerated mucus, capillary hyperplasia, and hemosiderin deposition (Figure 6). The pain and sensory disturbances completely resolved soon after surgery. Mild swelling at the resection site was observed at three months postoperatively but completely resolved after one year.

Case 2

**Figure 7:**
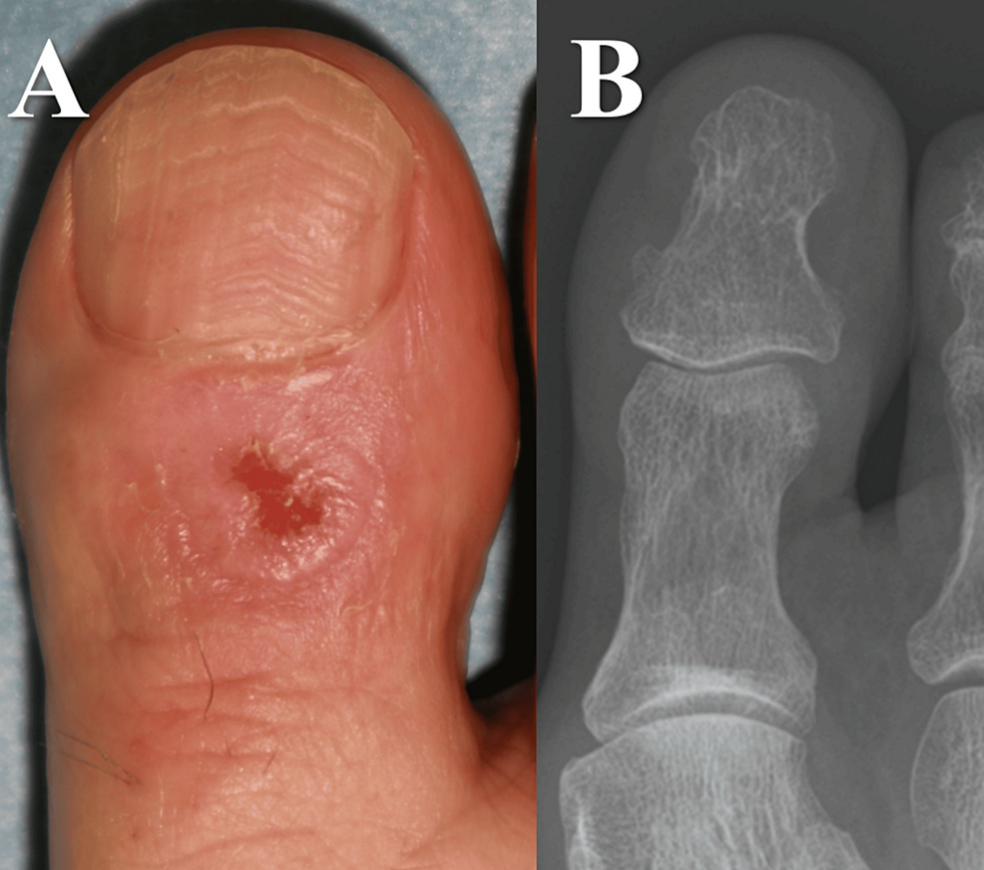
Preoperative clinical photograph and radiographic findings (Case 2) (A) A cyst was identified on the dorsal side of the IP joint. It appeared dry and shriveled. (B) Anteroposterior radiographs of the right hallux show no remarkable changes. IP: interphalangeal

A 69-year-old woman presented to our hospital with a 16-year history of dull pain in the dorsal aspect of her right hallux, attributed to a mass effect and occasional rupture. She had a ganglion cyst removed from the area 14 years earlier, but it recurred shortly thereafter. Repeated tumour crushing or puncture provided limited relief. The tumour shrank after the contents leaked out following rupture (Figure 7A). A radiograph showed no hallux abnormalities or osteoarthritic changes (Figure 7B).

**Figure 8:**
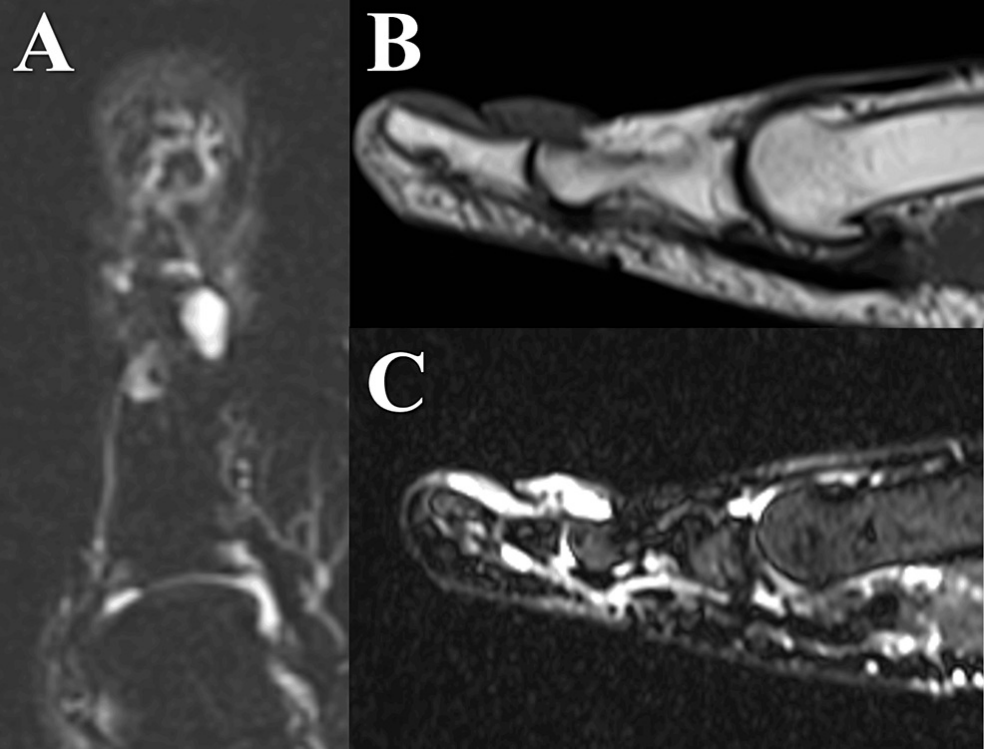
Findings on MRI (Case 2) A solitary cyst was found on the dorsal aspect of the interphalangeal joint. T1-weighted (B) and short tau inversion recovery (A-C) sequences showed fluid accumulations within the joint and the tendon sheath of FHL. MRI: magnetic resonance imaging, FHL: flexor hallucis longus

The MRI taken before the rupture revealed a 10 mm flat unifocal tumour that appeared hypointense on T1-weighted sequences and homogeneously hyperintense at the dorsal IP joint on short tau inversion recovery images. MRI also showed fluid accumulation within the joint and FHL tendon sheath (Figure 8).

**Figure 9:**
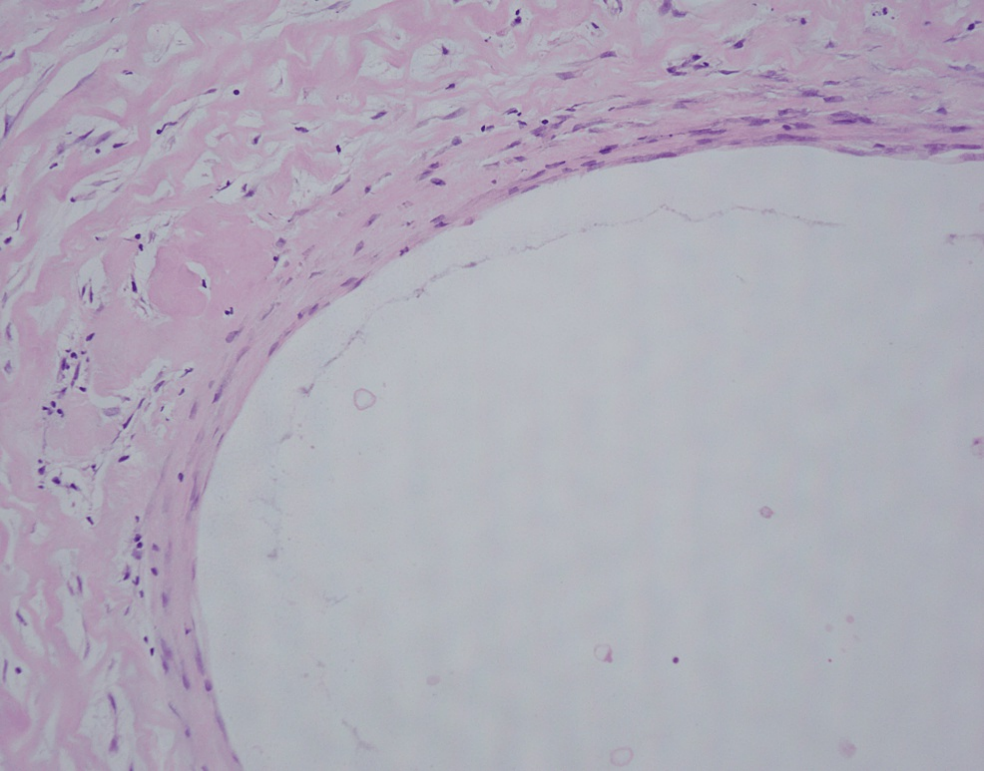
Histopathological analysis (Case 2) A ganglion cyst consists of a thin collagenous fibrous wall filled with degenerated mucus.

A spindle-shaped incision was made above the ganglion cyst. The cyst was communicating with the IP joint capsule, and cyst excision and synovectomy were performed. No fluid accumulation was found within the synovial sheath of the FHL during the incision made at the nearby cuneonavicular joint. No synovial fluid flowed into the cyst resection area when the plantar surface of the foot was compressed, indicating no continuity with the ganglion cyst. Histopathological analysis confirmed the diagnosis of ganglion (Figure 9), and no recurrence was observed during the one-year follow-up.

## Discussion

A ganglion is a benign cystic lesion containing gelatinous material that causes pain in about 50% of cases. It seldom leads to nerve damage from a mass effect on nearby nerves [1]. While ganglions in the foot account for only about 6.6% of all cases [2], they are often large and symptomatic [3]. Ganglion cysts around the hallux can cause severe pain, nerve damage, and self-destruction. The pathophysiology of hallux ganglions is not fully understood, but some have been found to originate from the FHL tendon sheath (as in Case 1) or the MTP/IP joint (as in Case 2). A hallux ganglion originating from the FHL tendon sheath mainly forms on the plantar side [2] and expands peripherally because of high internal pressure, reaching the distal end of the foot not only on the plantar side but also on the lateral and dorsal sides [5]. In contrast, a hallux ganglion originating from the MTP/IP joint tends to form on the dorsal side of the joint capsule [6]. Conservative measures include aspiration, manual rupture, steroid or hyaluronidase injections, and sclerotherapy. Surgical excision is recommended when a ganglion is resistant to conservative treatment and symptoms are severe.

Ganglion cysts result from weak spots in joint capsules or tendon sheaths, which bulge out and act as a check valve when pressure builds up from synovial fluid accumulation. Standard treatment involves dissecting and excising the valvular structure as deeply as possible [7], but recurrence rates are high (5% to 30%) [3]. One series reported a 43% recurrence rate after surgery for foot and ankle ganglions [8]. However, the main cyst is sometimes left in situ, and only the valve system is excised [9]. A hallux ganglion is constantly under pressure because of weight-bearing, and the cystic structure becomes incorporated into the surrounding tissue. Furthermore, ganglion cysts around the hallux have connections to the MTP/IP joint or synovial cysts in the FHL tendon sheath, known as communication stalks. One study found communication stalks in all cases of intractable hallux ganglion [2].

Incomplete removal of communication stalks is known to cause ganglion recurrence, highlighting the importance of understanding the cyst’s origin and its relationship with neighbouring joints to ensure complete excision of synovial fluid supply pathways.

Lee et al. reported that hallux ganglions originating from the FHL tendon sheath have a higher recurrence risk [2]. No recurrence was observed when communication stalks were excised during ganglion cyst removal and tendon sheath release.

The FHL passes through the base of the calcaneal tuberosity, travels in a groove on the posterior aspect of the calcaneus, passes through the plantar aspect of the sustentaculum tali, and intersects with the tendon of the flexor digitorum longus around the area of the tarsal tunnel. It then passes through the plantar aspect of the MTP and IP joints and terminates at the base of the distal phalanx of the hallux. The tendon sheath of the FHL is divided into proximal (from 1 cm proximal to the ankle joint to the tarsal tunnel) and distal (from the central part of the MTP joint to the distal phalanx) portions. This sheath is rarely continuous in normal cases [5].

Using ankle arthrography, Zhang et al. demonstrated communication between the ankle or FHL tendon sheath and the cyst in 13 of 19 patients [4]. They reported no recurrence after the removal of the ganglion cysts and ankle capsulorrhaphy, although the tendon sheath was not directly visualized. More cases are needed to determine the incidence of continuous pathological tendon sheath development.

In Case 1, ankle arthrography revealed fluid accumulation in the FHL tendon sheath in the posterior area of the ankle joint but no enhancement of the ganglion. Contrast injection into the ganglion showed no communication with the ankle. Furthermore, a large amount of synovial fluid was drained after the incision of the proximal FHL tendon sheath, but without a change in the tension of the ganglion. Therefore, the proximal and distal tendon sheaths were not considered to be continuous in this patient. Given that the hallux ganglion had communication with the distal FHL tendon sheath in this case, it is presumed that the cyst enlarged towards the distal end of the plantar aspect of the foot and that the valvular structure of the communication stalk was compressing the digital nerve. Mild swelling was observed at the resection site in the early postoperative period but was considered to represent a temporary accumulation of synovial fluid that flowed into the dead space after excision rather than recurrence. The sheath was released without a pressure increase, so fluid was assumed to have disappeared spontaneously. However, we believe that the ganglion in Case 2 originated in the IP joint despite swelling of the FHL tendon sheath, and decompression by excision of the joint capsule was successful. Although the cause of the intractable hallux ganglion remains unclear, we believe that healing was achieved by blocking the synovial supply and reducing pressure within the joint and tendon sheath.

## Conclusions

The risk of recurrence of an intractable hallux ganglion can be reduced by blocking the synovial supply route and lowering the pressure inside the joint or tendon sheath.
